# Supplementary figures and images for: Variation in TAF1 Expression in Female Carrier-Induced Pluripotent Stem Cells and Human Brain Ontogeny Has Implications for Adult Neostriatum Vulnerability in X-Linked Dystonia Parkinsonism
Source: eNeuro. 2022 Aug 17;9(4):ENEURO.0129-22.2022. doi: 10.1523/ENEURO.0129-22.2022 (PMC9428949; doi:10.1523/ENEURO.0129-22.2022)

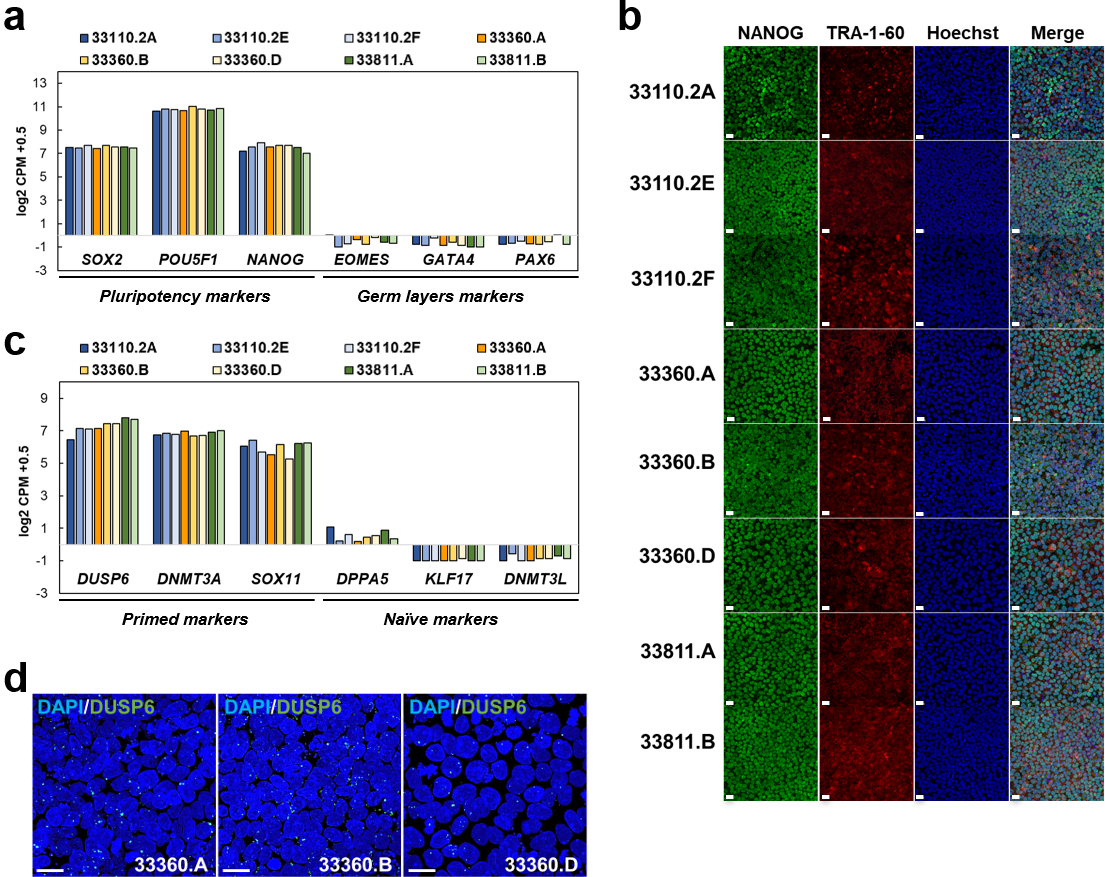

Supplement: Extended Data Figure 1-2 — Pluripotency characterization of XDP female carrier-derived iPSCs. a, Bulk RNA-sequencing analysis revealed that all XDP female carrier-derived iPSC clones expressed multiple markers associated with pluripotency, such as SOX2, POU5F1, and NANOG; they did not express genes associated with three germ layers: EOMES (mesoderm), GATA4 (endoderm), and PAX6 (neuroectoderm). b, Expression of the pluripotency markers NANOG (green) and TRA-1-60 (red) was detected by immunofluorescence staining in all eight XDP carrier iPSCs. Hoechst (blue) was used to visualize nuclei. Merged images depict overlays of immunoreactivity for each target, together with the nuclear counterstain. Scale bar: 20 μm. c, Comparative expression of primed (DUSP6, DNMT3A, SOX11) and naive (DPPA5, KLF17, DNMT3L) markers in all eight XDP female carrier-derived iPSCs. d, Representative images from isogenic XDP female carrier iPSC lines showing localization of RNA scope-specific probes for DUSP6 (green) transcripts. DAPI was used to stain nuclei. Scale bar: 20 μm. Figure contribution: Bareera Qamar performed the fluorescence immunostaining on iPSCs. Laura D’Ignazio performed the RNA scope assay. Kynon J. M. Benjamin performed the RNA-seq data analysis. Download Figure 1-2, TIF file. [file enu-eN-NWR-0129-22-s05.tif]

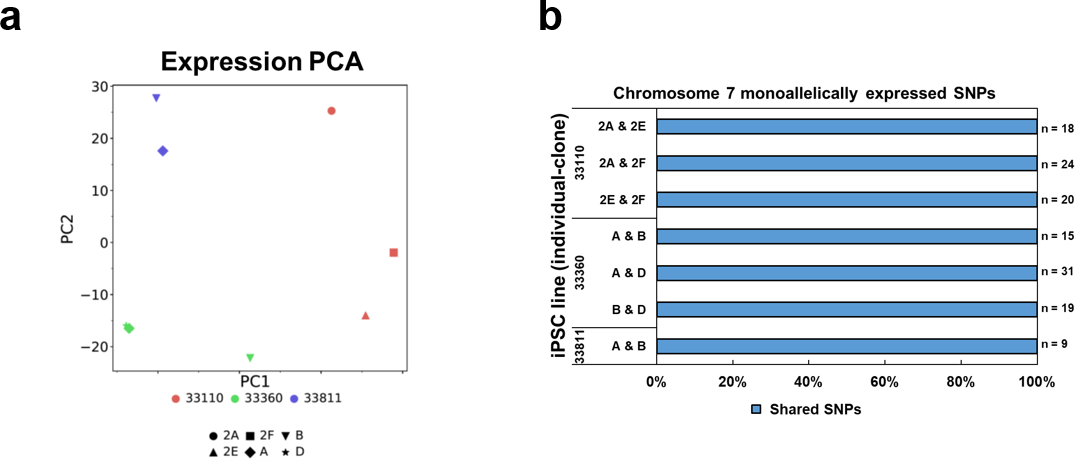

Supplement: Extended Data Figure 1-3 — XCI status of XDP female carrier-derived iPSCs. a, Principal component analysis of normalized expression of X-linked genes from the eight XDP carrier iPSC lines. b, Bar plots of the percentage of allele-specific transcripts derived from chromosome 7, which are shared between the indicated iPSC clones. Chromosome 7 was chosen as a reference autosomal chromosome because it has a similar size to chromosome X. Expression from the same or different alleles was determined by counting the number of shared and unique homozygous SNPs, respectively. The number of SNPs analyzed for each pair is shown at the right of each bar. Figure contribution: Kynon J. M. Benjamin performed the PCA analysis. Ricardo S. Jacomini and Apua C. M. Paquola performed the allele-specific transcriptomic analysis. Download Figure 1-3, TIF file. [file enu-eN-NWR-0129-22-s06.tif]

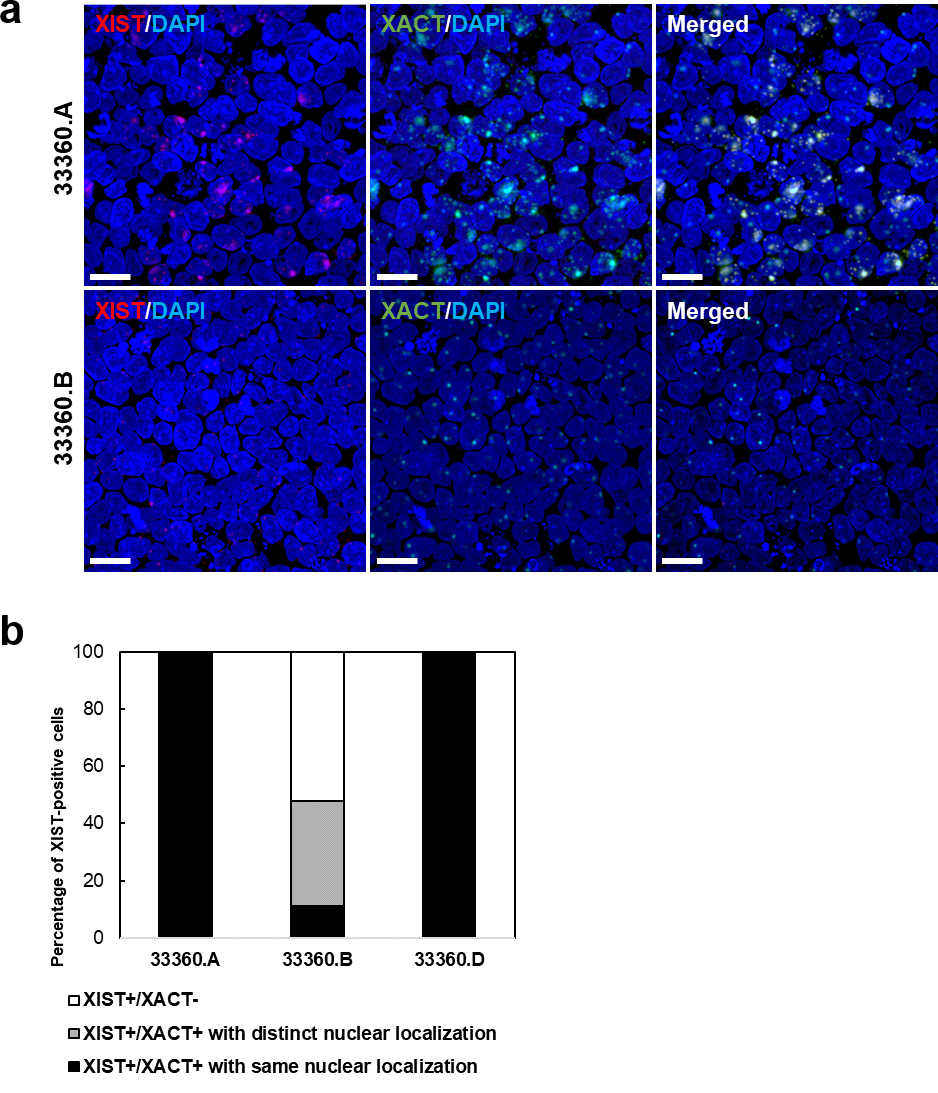

Supplement: Extended Data Figure 3-1 — XIST and XACT nuclear localization in XDP carrier-derived isogenic iPSCs. a, Representative images showing localization of XIST (red) and XACT (green) RNA scope probes in 33360.A and 33360.B iPSCs. DAPI was used to stain nuclei. Scale bar: 20 μm. b, Percentage of XIST-positive cells where XIST and XACT lncRNAs have distinct or shared nuclear localization in the set of isogenic XDP female carrier-derived iPSCs. For 33360.B, iPSCs also show the percentage of XIST+ cells not expressing XACT. Figure contribution: Bareera Qamar and Laura D’Ignazio performed the RNA scope assay and analyzed data. Download Figure 3-1, TIF file. [file enu-eN-NWR-0129-22-s07.tif]

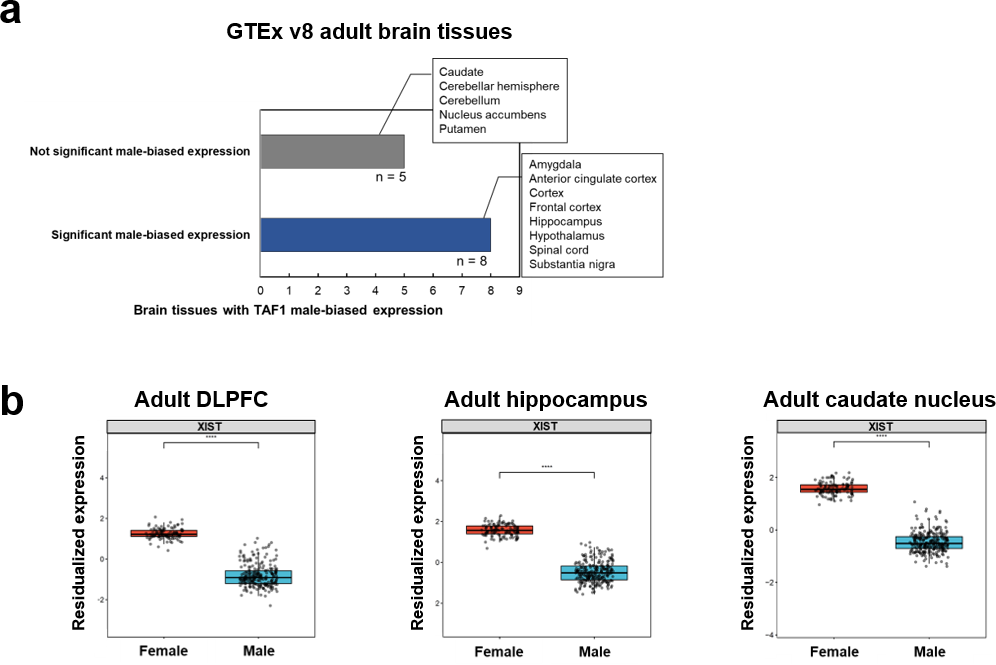

Supplement: Extended Data Figure 5-1 — Sex differential expression analysis of TAF1 and XIST across regions of postmortem adult brains. a, Number of postmortem brain tissues included in the GTEX v8 collection (Oliva et al., 2020) in which TAF1 expression is significantly or not significantly male biased. b, Box plots comparing residualized gene expression of XIST in female (red) and male (blue) individuals from the BrainSeq Consortium (Collado-Torres et al., 2019; Benjamin et al., 2020); caudate nucleus (n = 394), DLPFC (n = 379), and hippocampus (n = 376); ****p ≤ 0.001. Figure contribution: Laura D’Ignazio analyzed the publicly available GTEx dataset. Ria Arora and Kynon J. M. Benjamin performed the sex differential expression analysis. Download Figure 5-1, TIF file. [file enu-eN-NWR-0129-22-s08.tif]

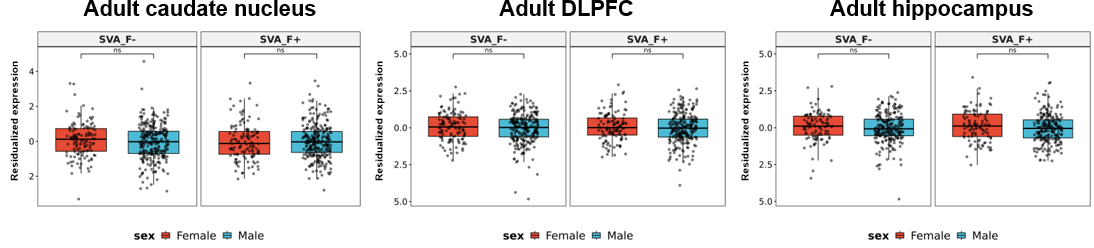

Supplement: Extended Data Figure 5-2 — SVA expression analysis in IPS lines and postmortem caudate nucleus. Box plots comparing residualized gene expression of subfamily F of the SVA retrotransposon in the caudate nucleus (n = 394), DLPFC (n = 379), and hippocampus (n = 376) of female (red) and male (blue) individuals from the BrainSeq Consortium (Collado-Torres et al., 2019; Benjamin et al., 2020); ns = not significant. Figure contribution: Taylor A. Evans performed the SVA-F differential expression analyses. Download Figure 5-2, TIF file. [file enu-eN-NWR-0129-22-s09.tif]
